# Comparative analysis of information contents relevant to recognition of introns in many species

**DOI:** 10.1186/1471-2164-12-45

**Published:** 2011-01-19

**Authors:** Hiroaki Iwata, Osamu Gotoh

**Affiliations:** 1 Department of Intelligence Science and Technology, Graduate School of Informatics, Kyoto University, Yoshida Honmachi, Sakyo-ku, Kyoto 606-8501, Japan; 2 National Institute of Advanced Industrial Science and Technology, Computational Biology Research Center, 2-4-7 Aomi, Koto-ku, Tokyo 135-0064, Japan

## Abstract

**Background:**

The basic process of RNA splicing is conserved among eukaryotic species. Three signals (5' and 3' splice sites and branch site) are commonly used to directly conduct splicing, while other features are also related to the recognition of an intron. Although there is experimental evidence pointing to the significant species specificities in the features of intron recognition, a quantitative evaluation of the divergence of these features among a wide variety of eukaryotes has yet to be conducted.

**Results:**

To better understand the splicing process from the viewpoints of evolution and information theory, we collected introns from 61 diverse species of eukaryotes and analyzed the properties of the nucleotide sequences relevant to splicing. We found that trees individually constructed from the five features (the three signals, intron length, and nucleotide composition within an intron) roughly reflect the phylogenetic relationships among the species but sometimes extensively deviate from the species classification. The degree of topological deviation of each feature tree from the reference trees indicates the lowest discordance for the 5' splicing signal, followed by that for the 3' splicing signal, and a considerably greater discordance for the other three features. We also estimated the relative contributions of the five features to short intron recognition in each species. Again, moderate correlation was observed between the similarities in pattern of short intron recognition and the genealogical relationships among the species. When mammalian introns were categorized into three subtypes according to their terminal dinucleotide sequences, each subtype segregated into a nearly monophyletic group, regardless of the host species, with respect to the 5' and 3' splicing signals. It was also found that GC-AG introns are extraordinarily abundant in some species with high genomic G + C contents, and that the U12-type spliceosome might make a greater contribution than currently estimated in most species.

**Conclusions:**

Overall, the present study indicates that both splicing signals themselves and their relative contributions to short intron recognition are rather susceptible to evolutionary changes, while some poorly characterized properties seem to be preserved within the mammalian intron subtypes. Our findings may afford additional clues to understanding of evolution of splicing mechanisms.

## Background

The intron is a nucleotide sequence that is transcribed from the genome but finally removed in the process of maturation of the transcribed RNA. The process of intron removal is called RNA splicing. Nuclear (or spliceosomal) introns are abundant in most eukaryotes and are processed by the machinery known as spliceosomes. In this article, we confine our attention to this type of intron. Other types of introns, known as Group I and Group II introns, are present in some archaea, bacteria, viruses, and plant and fungal organelles and are processed by auto-catalytic machineries [1]. Under normal circumstances, splicing occurs only between the 5' and 3' ends of the intron on the same transcript, although exceptional trans-splicing is also observed in some organisms [2]. The basic process of splicing is conserved among eukaryotic species. Three signals, the 5' splice site (5'ss), the 3' splice site (3'ss) and the branch point (BP), are commonly used to directly conduct splicing [3,4]. Another characteristic sequence is the polypyrimidine tract (PPT) that is located between BP and 3'ss, and is rich in pyrimidine bases. BP lies at less than dozens of nucleotides upstream of the PPT sequence. BP provides the means by which 3'ss is identified. In addition, broadly distributed exonic and intronic splicing enhancers/silencers are also involved in the correct recognition and regulation of a particular pair of 5'ss and 3'ss to be spliced. Besides these signal sequences that are directly involved in splicing, other features are also related to intron recognition, including intron length and nucleotide composition within the intron [5].

Although the basic ability of eukaryotes to splice introns has remained conserved throughout evolution [6-9], the splicing signals have considerably differentiated [10-12], eventually to the degree that the splicing mechanisms of remotely related organisms are incompatible with each other. For example, animal introns are not properly recognized in transfected plant cells [13,14]. Similarly, most mammalian introns are not recognized by the yeast *Saccharomyces cerevisiae *[15,16]. It is naturally hypothesized that the incompatibility is due to disparate changes in the splicing signals and their corresponding splicing factors. For example, PPT, which is recognized by U2AF^65 ^protein factor, shows highly variable lengths and signal strengths among species; although there is a certain bias for pyrimidines toward the 3' end of the intron among all organisms, this signal is weak among most fungi, stronger in plants and protozoans, and by far the strongest in vertebrates [17]. It is also known that the BP signal recognized by SF1 protein factor is highly conserved in yeast but variable in higher eukaryotes, although eukaryotes have a preference for purines or pyrimidines at each position and retain the target "A" nucleotide [6,18].

It is important to investigate the conservation and variation of features relevant to splicing throughout the evolution of eukaryotes from two points of view. First, it provides us fundamental knowledge of the evolution of this very complicated cellular process that involves many factors and their recognition sites. Second, the quantitative characterization of these features is indispensable for the development of accurate and efficient computational tools for gene recognition from the genomic sequence [19-21]. A few studies have been conducted along these lines [12,22] with a relatively small number of species. However, more comprehensive studies on a much wider range of eukaryotic taxa are now feasible as many sequences of complete or nearly complete genomes together with their transcripts have become available.

In this study, we characterized and analyzed splicing signals in 61 eukaryotic species. First, we analyzed the evolutionary relationships of individual splicing features. We found that the five evolutionary trees thus constructed (feature trees) roughly reflect the phylogenetic relationships among the species but sometimes extensively deviate from the species tree. Second, we measured the relative contributions of the five splicing features to recognize correct splicing pairs of short introns by means of a method similar to that used by Lim and Burge [5]. By comparing the relative contributions of these splicing features, we determined similarities in the short intron recognition pattern among the 61 species. The results again show that there is some correlation between the relationships thus estimated and the species tree, although great deviations are also often observed. These results indicate that the RNA splicing mechanism is rapidly evolving in some lineages. When confined to mammals, the overall pattern of intron recognition is well conserved. In particular, we found that the GT-AG, GC-AG, and AT-AC subtypes of introns form independent categories among all the introns in mammals.

## Results

### Basic characteristics of intron data

**Figure 1:**
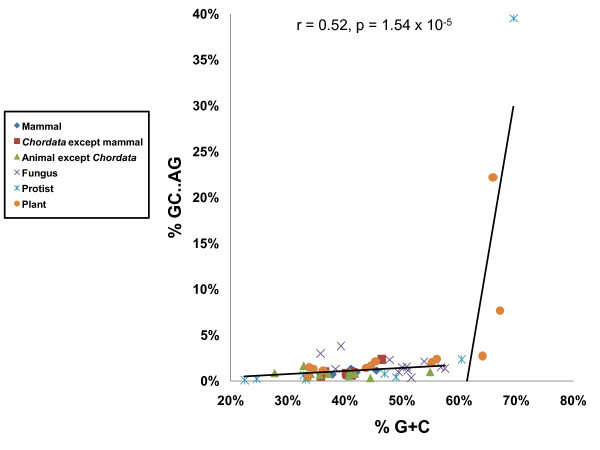
**The correlation plot between genomic G + C content and the fraction of GC-AG introns**. The right regression line was obtained from the data of the five species with the highest genomic G + C contents, while the left regression line was derived from the data of the other species.

We classified introns into three subtypes according to their terminal dinucleotide sequences. GT-AG introns represented 98.6%, GC-AG introns, 1.3%, and AT-AC introns, 0.1% of ca. 2.46 × 10^6 ^introns examined. However, these fractions show considerable variation among organisms (see Additional file 1). The fractions of GC-AG introns in *Aureococcus anophagefferens *(alga) and *Micromonas pusilla *(picophytoplankton) are exceptionally high, respectively accounting for 39.6% and 22.2% of all introns of the species. The high G + C contents of the genomic sequences of these species may be related to these observations. In fact, the genomic G + C content of *A. anophagefferens *is 69.5% and that of *M. pusilla *is 65.9%, which are the highest and the third highest among the 61 species examined, respectively. The fraction of GC-AG introns in *Chlorella sp*. with the second highest genomic G + C content is 7.7% and the third highest among the 61 species. Thus, it seems apparent that the high genomic G + C content is responsible for the high fraction of GC-AG introns. However, there must be other factors that affect the abundance of GC-AG introns, as the correlation between the genomic G + C content and the fraction of GC-AG introns is rather weak except for these three species as shown in Figure 1. Moreover, the GC-AG fraction (2.7%) of *Micromonas RCC299*, another picophytoplankton, is not as remarkable as that of *M. pusilla *despite their close taxonomic relationship and its relatively high genomic G + C content (64.1%).

The fraction of AT-AC introns is small in all species. Although *Caenorhabditis elegans *seems to lack snRNAs U11, U12, and U4atac that comprise U12-type spliceosomes [23], our automated procedure (see Methods) identified 59 AT-AC introns in *C. elegans *(Additional file 1). By manually inspecting the alignments, we suspect that at least 42 of them are real AT-AC introns, while the others can be non-canonical ones. The observed AT-AC introns are probably processed by U2-type spliceosomes in this species.

We aggregated intron length data separately for each species and for each of the three subtypes of introns. However, no significant difference was observed in intron lengths categorized into the two subtypes of GT-AG and GC-AG in each species. As the contributions of AT-AC type introns are negligible, we used the combined data in the following analyses. More primitive organisms tend to have shorter average lengths of introns, as has been often documented [24-26]. *Homo sapiens *has the longest (6751 bp) average intron length and fungal *Phanerochaete chrysosporium *has the shortest (56 bp) one among the 61 species. When sorted by average intron length in descending order, all highly ranked species are occupied by animals (Mann-Whitney *p *= 0.18 × 10^-8^), in particular, by mammals within animals (Mann-Whitney *p *= 0.20 × 10^-6^). By contrast, lower ranked species are mostly fungi (Mann-Whitney *p *= 0.26 × 10^-5^).

Additional file 2 shows the percentage of introns having PPT, the average PPT length, and the percentage of pyrimidine nucleotides in PPTs for each species (see Additional file 3 for numerical values). PPTs are detectable in more than 99% of mammalian introns. Both average PPT length and C + T content within PPT are well conserved among mammals: the average PPT length is approximately 18 bp and the average C + T content is approximately 87%. Mammals have the longest PPT and the highest C + T content within PPT among the six groups defined in Methods. The general features are conserved in other vertebrates, but become gradually divergent when we look at other metazoan species. Outside animals, particularly in fungi and protists, PPTs tend to shrink and have relatively low average C + T contents.

**Figure 2:**
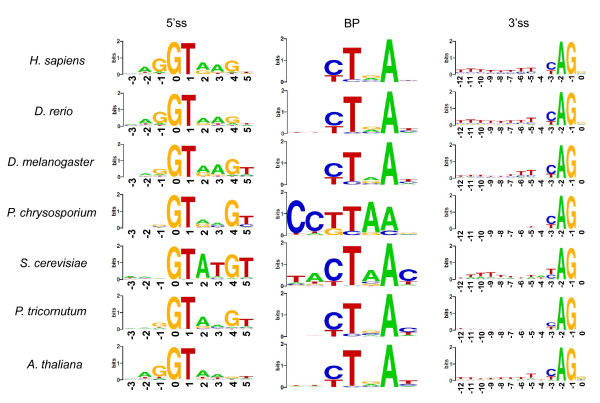
**Splicing signal motifs of seven species**. Sequence motifs for 5'ss, 3'ss, and BP are depicted by Sequence logos WebLogo http://weblogo.berkeley.edu/. The relative height of each letter is proportional to the relative entropy of the corresponding base at the given position, and bases are listed in descending order of frequency from top to bottom.

We analyzed the classical splicing signal motifs for each organism. The results of seven representative species from six groups, *H. sapiens*, *Danio rerio*, *Drosophila melanogaster*, *P. chrysosporium*, *S. cerevisiae*, *Phaeodactylum tricornutum*, and *Arabidopsis thaliana *shown in Figure 2 reveal well-known motif profiles. Although resembling one another, the motif profiles exhibit some specificity. For example, the preference of G for the + 4 position of 5'ss is very strong in fungi but marginal in plants. Moreover, the motif profile of BP is highly conserved in *S. cerevisiae *and *P. chrysosporium *but less remarkably conserved in the other species. In this way, the motif profiles are characteristic to species or groups.

**Figure 3:**
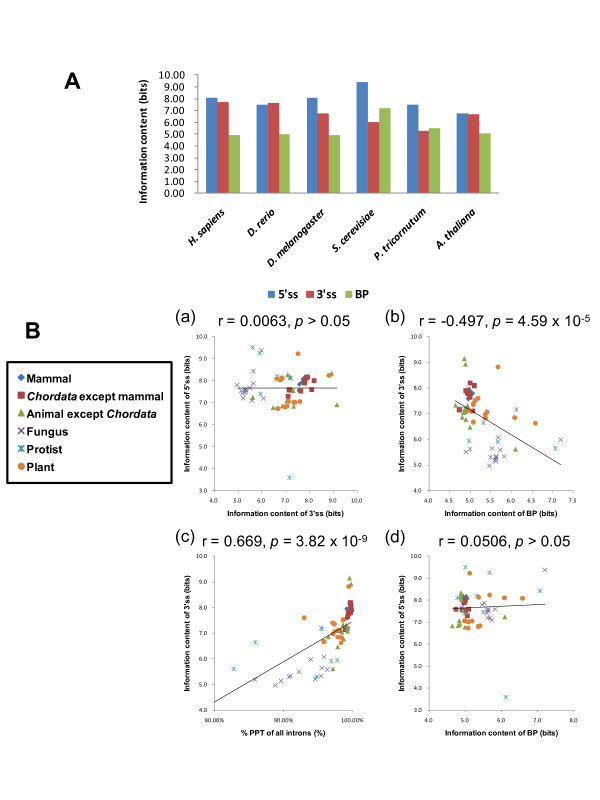
**Information contents and correlations of three splice signal motifs**. (A) The information contents of 5'ss (blue), 3'ss (red), and BP (green) motifs are measured in bits. Results for only representative species are shown here. Complete numerical data are presented in Additional file 4. (B) Correlation between (a) information contents of 3'ss and 5'ss, (b) information contents of BP and 3'ss, (c) % PPT of all introns and information content of 3'ss, and (d) information contents of BP and 5'ss. 61 species are categorized into six groups, as shown in the legend on the left. The Pearson correlation coefficient and the significance thereof are shown on top of each plot.

We also calculated the information contents of the motif profiles of all species as described in Methods, and summarize the results in Additional file 4. From the viewpoint of information contents, the same tendency as that in the motif profiles could be observed. For example, in mammals, the information contents of 3'ss are high but those of BP are low, whereas the inverse applies to fungi (Figure 3A). The information content of BP of *S. cerevisiae *is the highest of all the 61 species. When examined for all the 61 species, the information contents of 3'ss and BP show a strong negative correlation, whereas those of 3'ss and the percentage of PPT-containing introns show a strong positive correlation (Figure 3B). These observations are in good agreement with the qualitative pattern of the motif profiles shown in Figure 2.

### Trees of 61 species constructed from individual intron features

**Figure 4:**
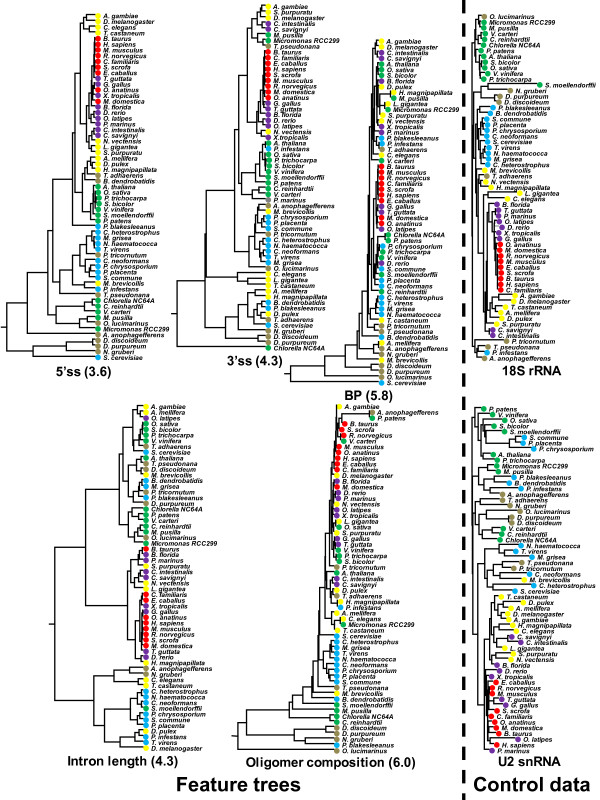
**Five feature trees and reference trees for the 61 species**. Mammal (red), *Chordata *except mammal (purple), animal except *Chordata *(yellow), fungus (blue), protist (beige), and plant (green). The number in parentheses indicates the mean of RMSD values between the feature tree and the reference trees derived from multiple sequence alignments of 18S rRNAs and U2 snRNAs. The means and standard deviations of the RMSD values obtained from the random tests with 100 trials and the *p*-values estimated there from are as follows. 5'ss: 6.0 ± 0.1 (*p *= 1.4 × 10^-127^), 3'ss: 6.1 ± 0.2 (*p *= 1.1 × 10^-19^), BP: 6.3 ± 0.2 (*p *= 6.2 × 10^-3^), intron length: 5.6 ± 0.1 (*p *= 6.1 × 10^-39^) and oligomer composition: 6.9 ± 0.2 (*p *= 3.4 × 10^-6^).

We took up five features that are related to intron recognition as mentioned in Methods. For each feature, we calculated the distance between a pair of species and built a dendrogram (feature tree) of the 61 species using those distances (Figure 4). As stated in the previous subsection, considerably variable motif profiles could be observed for individual species. We naturally suspected that each lineage has a similar tendency with respect to a particular feature. This is in fact the case for the nine mammalian species; the mammalian species occupy nearby positions in all the five feature trees. The concordance between species tree and feature tree is most prominent for 5'ss, representing nearly monophyletic appearances among various animal phyla, among land plants, and among fungi. This tendency gradually weakens for 3'ss, intron length, BP, and oligomer composition in this order. Remarkably, the feature tree for intron length does not show monophyletic topology even among mammals, suggesting the rapid evolutionary change of this feature.

### Feature trees of mammalian species

For the nine mammalian species, we classified the introns into three subtypes according to their terminal dinucleotide sequences. This is feasible as considerably large amounts of data are available for most of the nine mammalian species. We then made profiles for individual subtypes and features, calculated the distance matrices, and constructed a dendrogram for each feature. As the number of AT-AC introns is small, we could not fit the observed length distribution of AT-AC introns to a superposition of two Frechet distributions [27]. Hence, we did not construct the feature tree for length distributions of AT-AC introns.

**Figure 5:**
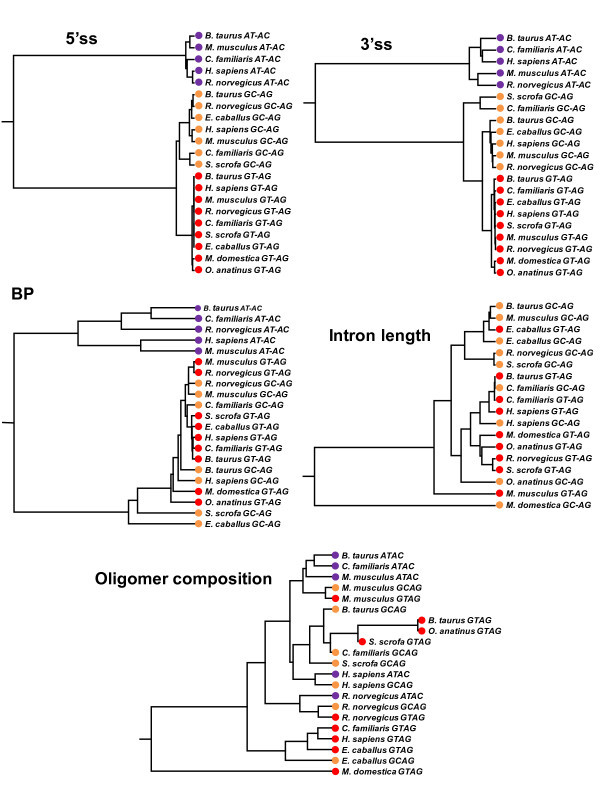
**Feature trees constructed from mammalian intron data**. Taxa of each feature tree are categorized into subtypes according to the terminal dinucleotides of the introns, GT-AG (red), GC-AG (orange), and AT-AC (purple). For statistical reasons, only intron data with GT-AG and GC-AG, and other data with more than 100 instances are used for this analysis.

As shown in Figure 5, GT-AG, GC-AG, and AT-AC introns form respectively monophyletic groups in the feature tree for 5'ss across the mammalian species. Note that the difference in the terminal dinucleotides was disregarded in calculation of the distances. A similar topology was also observed in the 3'ss profiles although GC-AG introns are not perfectly monophyletic. It is rather surprising that GT-AG and GC-AG introns form separate quasi-monophyletic groups, albeit their sharing the same 3' terminal dinucleotides. This observation probably reflects the mechanism in which 5'ss and 3'ss cooperate to find specific counterparts in the process of splicing.

It is obvious that AT-AC 5'ss and 3'ss boundary signals are clearly distinct from those of the other subtypes. This difference is certainly related to the fact that AT-AC introns are mainly processed by U12-type spliceosomes, whereas GY-AG introns are mainly processed by U2-type spliceosomes. It is reported that U12-type spliceosomes can also process GY-AG introns [28] and conversely, U2-type spliceosomes can potentially process AT-AC introns. Although some operational rules were proposed to discriminate U12-type and U2-type introns based on sequence characteristics [29], we did not distinguish U2-type and U12-type introns, as we considered that more experimental verification would be needed to apply the rules to a large volume of data obtained from diverse species. We will return to this issue later in Discussion.

The feature tree for BP motif shows that AT-AC introns form a monophyletic group, while GT-AG and GC-AG introns are not clearly separated from each other. This observation is consistent with the report that U12-type introns possess a well-conserved BP motif [11].

For the other feature, oligomer composition, the terminal dinucleotide types are even less correlated with the branching patterns of the feature trees. For oligomer composition, the same species rather than the same terminal dinucleotide type tend to make a cluster.

The above observations suggest that GT-AG, GC-AG, and AT-AC introns comprise distinct functional groups that are conserved among various mammals. The 5'ss and 3'ss signals are most influential and BP may also be involved in the functional differentiation. In contrast, the contribution of the other features is minimal.

### Relative contributions of intron features to short intron recognition

**Figure 6:**
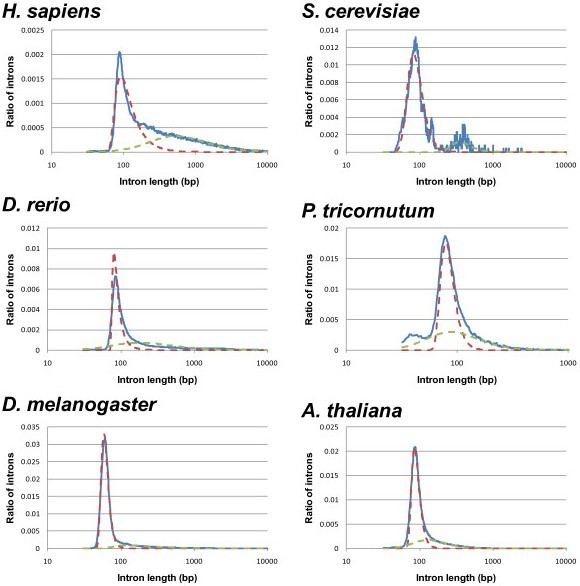
**Length distributions of all introns from six species**. The blue solid line shows the observed length distribution. The dashed lines (red and green) show individual components of two Frechet distributions fitted to the observed distribution with the maximum likelihood method.

To study the individual contributions of various intron features, we examined only short introns, because long introns are likely to be recognized by much more complicated mechanisms than the five features considered here. For example, exonic and intronic splicing enhancers/silencers, secondary structure of pre-mRNA, and epigenetic modulation of coding genes are likely to be involved in exon and intron definition mechanisms [30,31] but are out of consideration in this investigation. The histograms of intron lengths revealed the presence of distinct populations of short introns in individual organisms. By fitting the observed length distribution to a superposition of two Frechet distributions [27], we might be able to determine the natural cutoff length for short introns for each species (Figure 6). However, the varying cutoff lengths would introduce additional variability to our analysis and hence, we preferred to use a fixed value as the threshold of short and long introns. To determine the threshold, we averaged the short intron distributions of the 61 species and defined the threshold as 250 bp, which is the 95% quantile of the average distribution. The total numbers of short introns determined by this procedure are listed in Additional file 1. The fraction of introns classified as short is approximately 15% in mammals. By contrast, high fractions exceeding 80% are often found in fungi and protists.

To analyze the contributions of the five features to the recognition of short introns, we employed a variant of the method of Lim and Burge [5] (see Methods). Note that the features used for the analysis were obtained not only from short introns but from all introns of a species. In this analysis, the amount of contribution of each feature is estimated from the gain in intron recognition accuracy achieved by that feature. Additional file 5 shows the amount of contribution of each feature in each species and Additional file 6 shows the absolute and fractional values measured in bits in each species. The sum of the relative contributions of 5'ss and 3'ss exceeds 50% in most species. The contribution of BP is generally low. The contribution of intron length is also generally low except for fungi and plants, which have significantly higher contributions than the other groups (Mann-Whitney *p *= 0.58 × 10^-4^). *A. anophagefferens *has the highest fraction (over 45%) of information deficit among the species. By contrast, the fraction of information deficit is the smallest (approximately 7.3%) in *Dictyostelium discoideum*. When the 61 species are sorted by the information bit score of the information deficit, the nine mammals and the 15 vertebrates are ranked high (Mann-Whitney *p *= 0.17 × 10^-3 ^and *p *= 0.61 × 10^-5^, respectively), indicating that the intron splicing of higher eukaryotes requires more information other than the five features, compared with splicing in the other species.

**Figure 7:**
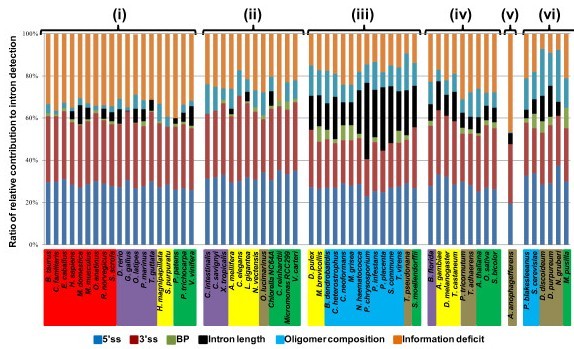
***K*-means clustering analysis of contributions of the five features to intron recognition**. Mammal (red), *Chordata *except mammal (purple), animal except *Chordata *(yellow), fungus (blue), protist (beige), and plant (green).

As the relative contributions of the features including information deficit vary considerably among species, we conducted cluster analyses of the patterns of these contributions among the 61 species. We tried the *k*-means clustering method. Prior to applying this method, we used principal component analysis (PCA) to obtain the first and second principal components (Additional file 7), and then ran self-organization map (SOM) on this coordinate system. As the SOM resulted in six major clusters (data not shown), we specified six as the number of clusters in the *k*-means clustering method. Figure 7 shows the results of the *k*-means clustering method applied to the 61 species. In cluster (i), the ratio of information deficit is large, amounting to nearly 40%. Concomitantly, the fractions of BP, intron length, and oligomer composition are relatively small. Cluster (ii) resembles cluster (i) except for the significant contribution of oligomer composition (ca. 10%). In cluster (iii), the contribution of intron length is considerably large while that of information deficit is relatively small. Cluster (iv) is rather uncharacteristic; here, the five features moderately contribute to intron recognition, leaving an information deficit of approximately 20%. Cluster (v) is the smallest cluster and is composed of only one species, *A. anophagefferens*. The ratio of information deficit is the largest of the six clusters. This is probably related to the unusually high fraction of GC-AG introns found in this species as mentioned earlier in this section. In the last cluster (vi), the contribution of oligomer composition is the largest and the percentage of information deficit is the smallest among the six clusters.

Of the six *k*-means clusters, some are well characterized by specific groups of species. For example, cluster (iii) consists of 14 species, 10 of which are fungi, while two fungal species *Phycomyces blakesleeanus *and *S. cerevisiae *are outside this cluster. We also found that all vertebrates except *Xenopus tropicalis *belong to cluster (i), and all *Chordata *except *Branchiostoma florida *belong to cluster (i) or cluster (ii). Likewise, five land plants belong to cluster (i) or cluster (iv); *Populus trichocarpa *and *Vitis vinifera *are in cluster (i), and *Sorghum bicolor*, *Oryza sativa*, and *A. thaliana *are in cluster (iv). Thus, evolutionarily close species tend to use similar strategies for intron recognition.

However, it should also be noted that every cluster except cluster (v) consists of more than one group of species of distant evolutionary origins. For example, clusters (i), (ii), (iii), and (v) individually contain both animal and plant species. From the distribution of plant species over the clusters, we speculate that plants have adopted divergent strategies for intron recognition in the course of evolution.

## Discussion

This work involves large-scale computational analysis of pre-mRNA splicing that has been enabled by taking advantage of recent progress in massive genomic and transcriptomic sequencing efforts. Our analysis is unique in that it uses only experimentally verified, high quality, and least redundant data collected by the use of our own mapping and alignment tool, *Spaln *[32]. Note that *Spaln *is one of the most accurate alignment tools currently available [33], and reportedly outperforms tools that are used for annotation in major databases, such as NCBI [34] or Ensembl [35].

Our observations are largely consistent with and reinforces those reported previously [5,12] with respect to the following points.

First, the five features that are involved in intron recognition are differentially conserved in the evolution of eukaryotes; 5'ss and 3'ss motifs are better conserved than BP, intron length, and oligomer composition. On one hand, this is revealed by the larger values of the information contents of 5'ss and 3'ss motifs than those of BP (Figure 3) and the other features. On the other hand, this tendency is also shown by the persistence of individual features across various species. To measure the degree of evolutionary persistence of the five features, we built a dendrogram using the distances between species with respect to each of the five features, and then evaluated how much the feature trees deviate from the species classification. As shown in Figure 4, the information contents of the motifs that reflect intra-species conservation are well correlated with the inter-species evolutionary persistence of the corresponding features. In this study, we used phylogenetic trees derived from 18S rRNAs and U2 snRNAs as reference. Although we also examined U1 snRNA sequences, the results are omitted here as the structures of fungal U1 RNAs extremely deviate from those of the other groups [36].

Second, PPT signals and BP signals compensate each other. Typically, mammals have strong PPT signals and weak BP signals, while the opposite is true in fungi. This compensating effect is further confirmed in other phyla of eukaryotes, as revealed by the strong negative correlation between PPT and BP signals (Figure 3). Like Schwartz and Silva et al. [12], we also found a gradual increase in PPT signal strength along the metazoan lineages (Additional file 2); protists and fungi have short PPTs and low C + T contents in PPT, whereas mammals have the longest PPTs and the highest C + T contents in PPT among the six groups. Insects and *Chordata *other than mammals have intermediate strengths with regard to these properties. In protists, PPTs are short but C + T contents are high, whereas PPTs are long but C + T contents are low in plants. Thus, PPT signals are ubiquitous but evolutionarily variable in both strength and constitution.

Third, some correlation is observed between the relative contributions of the splicing features to intron recognition and the genealogical relationships among the species. Typically, mammals and vertebrates exhibit similar patterns. However, the correlation seems to be valid for relatively small groups of species. We evaluated the contribution of each feature with a variant of the method of Lim and Burge [5]. Our method differs from that of Lim and Burge in two major points: (i) choice of the threshold between short and long introns, and (ii) collection of false exon-intron junctions. These modifications are necessary to automate large-scale comparisons with as little bias and noise as possible. The large average number of false junctions (about 400) relative to a true one tends to complicate our examinations, which leads to relatively large fractions of information deficits. Moreover, some of the false junctions may actually be real ones if alternative splicing is taken into account. Thus, there remains considerable room for improvement in the quantitative evaluation of the relative contributions of various features to intron recognition.

For the nine species of mammals, we classified the introns into three subtypes according to their terminal dinucleotide sequences. We constructed feature trees for each of the three subtypes of introns. Figure 5 shows that GT-AG, GC-AG, and AT-AC introns form respectively monophyletic groups in the feature tree for 5'ss. The feature tree for 3'ss also shows quasi-monophyletic groups between GT-AG and GC-AG introns. These results indicate that in RNA splicing, GT-AG and GC-AG introns use distinct 5'ss and 3'ss motif profiles despite the identical 3' end dinucleotides "AG". AT-AC 5'ss and 3'ss boundary signals are clearly distinct from those of the other subtypes.

**Figure 8:**
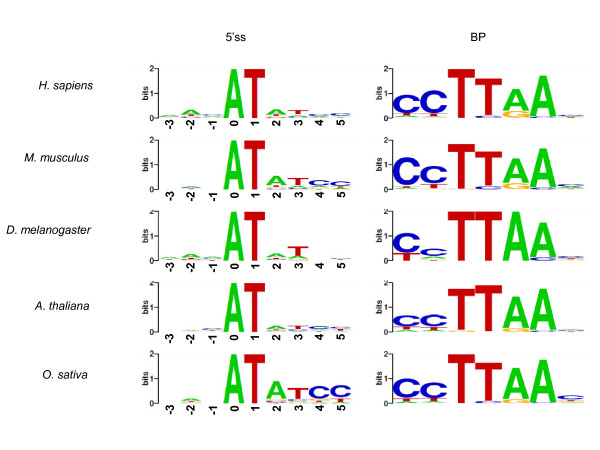
**5'ss and BP motif profiles of all AT-AC introns in five representative species**. The reported consensus sequences of U12-type 5'ss signal and BP are "ATATCC" and "CCTTAAC," respectively.

**Figure 9:**
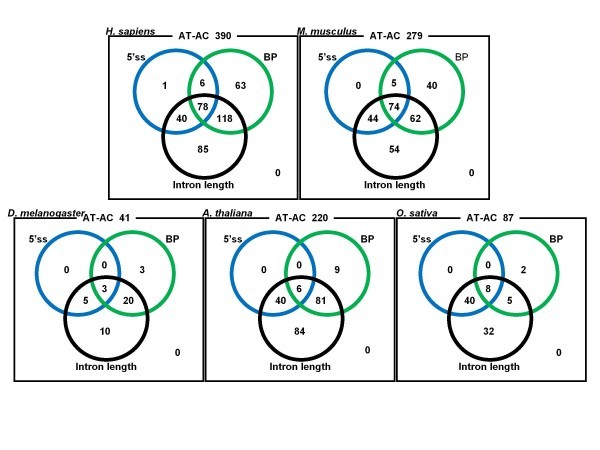
**Venn diagrams of AT-AC introns that satisfy the three criteria**. Each circle represents the fraction of AT-AC introns that satisfy one of the three U12-type criteria described in the text.

AT-AC introns are generally considered to be the U12 type [10]. To better characterize this minor type of introns, we investigated AT-AC introns in five species, *H. sapiens*, *Mus musculus*, *D. melanogaster*, *A. thaliana*, and *O. sativa*, whose genomic sequences are of high quality. Figure 8 shows 5'ss and BP motif profiles constructed from only AT-AC introns in these species. The observed motif profiles of 5'ss for the five species, in particular *O. sativa*, are consistent with the previously reported consensus sequence of U12-type introns "ATATCC" [28,37,38]. The motif profiles of BP even better match the reported consensus sequence of U12-type introns "CCTTAAC" [10]. We employed the weight matrix approach [11,37] to identify U12-type introns among AT-AC introns. The empirically derived rules were (i) 5'ss motif scores > 9 bits, (ii) BP motif scores > 6 bits, and (iii) intron lengths < 20 kb, where the weight matrix was constructed from a set of 48 experimentally verified U12-type introns [28]. The results summarized in Additional file 8 somewhat underestimate the frequencies of AT-AC U12-type introns relative to those of all introns, compared with the results of Sheth and Roca et al. [11]. In our analysis, more than half of the AT-AC introns satisfied the BP motif criterion in human, while only approximately 30% of the AT-AC introns satisfied the 5'ss motif criterion. Similar tendencies were also observed for the other species except *D. melanogaster *(Figure 9 and Additional file 8). We speculate that the threshold of 9 bits for the 5'ss motif score is too high, and the fraction of U12-type introns among AT-AC introns would actually be larger than the present estimates. However, more detailed experimental studies would be necessary to confirm this idea.

The origin of the exceptionally abundant GC-AG introns in *A. anophagefferens *and *M. pusilla *is an enigma. To see some peculiarity in the splicing machinery of these species, we retrieved putative snRNA U1 genes from the genomic sequences of these two species and another picophytoplankton, *M. RCC299*, as reference. Despite the high overall genomic G + C contents, *A. anophagefferens*, *M. pusilla*, and *M. RCC299 *putative U1 RNAs have G + C contents of 52.7%, 57.4%, and 58.4%, respectively, which are comparable to the average (55.5 ± 3.8%) of 100 U1 RNAs represented in the Rfam RF00003_seed alignment. The three putative U1 RNAs have the same 5'-terminal 11-nucleotide sequence that is perfectly identical to the consensus "AUACUUACCUG" that is directly involved in base pairing with the pre-mRNA donor site [39]. *M. pusilla *and *M. RCC299 *putative U1 RNAs exhibit 82.2% nucleotide identities in their entire ranges of 162 and 161 nucleotides, and these two sequences are equally divergent (approximately 55% identities) from the *A. anophagefferens *sequence. Altogether, it is unlikely that the very different fractions of GC-AG introns, 22.1% and 2.71%, in the two picophytoplanktons could be ascribed to the difference in the primary sequences of U1 RNA. Either RNA base modifications or the protein components of the U1 snRNP complex may be responsible for the peculiar 5' intron end selection in *A. anophagefferens *and *M. pusilla*.

## Conclusions

We have characterized representative features involved in RNA splicing in divergent eukaryotic species from the viewpoints of evolution and information theory. The quantitative characterization of these features is indispensable for the development of accurate and efficient computational tools for gene recognition of various types, such as *ab initio*, transcript-dependent, and comparative genomes, from the genomic sequence. As generally conceived and confirmed in this study, the 5'ss and 3'ss signals are the most important for the recognition of introns. Therefore, in many tools, exon-intron boundaries are recognized by position-specific weight matrix (PWM) or similar methods [40-42]. Our results indicate that the contributions of intron length and oligomer composition are also significant to varying degrees depending on the species or group. For example, Additional file 5 indicates that intron length considerably contributes to intron recognition in fungi and protists. In the latter, the contribution of oligomer composition is also comparable to that of intron length. Hence, a gene-finding program would benefit from incorporation of these features with an appropriate set of parameters adjusted for each species to achieve better performance.

However, it must be said that the present study has posed additional puzzles about the splicing mechanisms. Namely, the nearly monophyletic distribution of GT-AG, GC-AG, and AT-AC subtypes among mammalian introns with respect to 5'ss and 3'ss signals and the extraordinarily abundant GC-AG introns in some species are not easily explained by our present knowledge. Our results also suggest that U12-type introns may account for a larger fraction of AT-AC introns than existing estimates [11]. More detailed experimental studies on major- and minor-type introns would be necessary to reveal the mechanisms underneath these phenomena.

## Methods

### Data set

The complete genomic sequences of the 61 species were downloaded from NCBI http://www.ncbi.nlm.nih.gov/, Ensembl http://www.ensembl.org/ or Joint Genome Institute (JGI) http://genome.jgi-psf.org/. The "unique" set of Unigene data of each species was downloaded from NCBI [43]. EST sequences in JGI were also downloaded when available. For convenience, we divided the 61 species into six groups: mammal, *Chordata *except mammal, animal except *Chordata*, fungus, protist, and plant.

We used *Spaln *[32] to align Unigene and, if available, additional EST sequences to the genomic sequence of the same species. *Spaln *can map exon-intron structures quite accurately [32]. Using the alignment results, intron and flanking exon sequences were extracted from the genomic sequence. To obtain only reliable introns, we applied the following relatively stringent criteria for the quality of the alignment. (i) The intron ends must follow the canonical rule, *i.e.*, they must be GT-AG, GC-AG or AT-AC. (ii) The intron must be longer than 30 bp. (iii) The alignment flanking the intron must contain at most one mismatch and no gap within the ten nucleotide positions at each side. Moreover, to remove redundancy, we randomly selected only a representative intron when several exon-intron boundaries shared highly similar sequences (> 75% identities within a range of 100 nucleotides, half from the exonic and the other half from the intronic regions). Thus, the introns in our study are all evidence-based, of high quality, and the least redundant.

In analysis of information content and construction of feature trees, we used all intron which we detect. On the other hand, in analysis of evaluation of relative contribution of five features, we used only short intron (under 250 bp).

### PPT prediction algorithm

We devised an algorithm to find the most probable location of PPT within 50 nucleotides upstream of 3'ss. First, we identified the end (3' most) point of PPT as the last pyrimidine dinucleotides upstream of 3'ss. Then, we scanned the nucleotide sequence from there toward the 5' direction to compute the *PPT_score*, which is defined as the sum of the following indices:

(1) $$PPT\_index=\{\begin{array}{cc} +1.0 & \text{if }\left(\text{C or T}\right)\\ -1.5 & \text{if }\left(\text{A or G}\right) \end{array}$$

We stopped the scan when the *PPT_score *decreased by 2 or more from the maximum value so far attained, and identified the start (5' most) point of PPT as the position with the maximal *PPT_score*. When the percentage of C + T nucleotides inside the sequence is less than 50%, we regarded that the intron lacks PPT.

### BP prediction algorithm

To construct the BP signal profile, we developed a simple algorithm that extracts putative BP motifs from the introns of individual organisms. Assuming that a functional BP must be followed by a functional PPT of a certain length, we first scanned upstream of the PPT start point and located all possible BP sequences within 100 nucleotides upstream of 3'ss. In this scoring process, we used the "core" BP motif profile represented by a 4 × 5 PWM [44] to obtain the core BP score, *BP_score*(*i*), at the genomic position *i*. The detailed procedures for constructing PWM and calculating an associated score will be described later in this section. If the maximal *BP_score*(*i*) exceeds 1.85 bits [22], we regarded this 5-mer plus the upstream 2-mer as the BP signal of the intron. By repeating this procedure for all introns of each organism extracted as described above, we constructed the BP motif profile of the organism represented by a 4 × 7 PWM.

### 5'ss and 3'ss motif profiles

We constructed the motif profiles for each species, using all the extracted introns of the species. We first obtained the gap-less alignment around each splicing junction juxtaposing the canonical dinucleotide of 5'ss (GY or AT) or 3'ss (AG or AC). Looking at the relative entropy at each aligned position calculated with the overall genomic nucleotide compositions as the background, we selected informative signal sequences for each of the 5'ss and 3'ss motifs. Specifically, we identified positions -3 to + 6 from the exon-intron junction (exonic 3 positions and intronic 6 positions) as the 5'ss motif, and positions -13 to + 1 from the intron-exon junction (intronic 13 positions and an exonic position) as the 3'ss motif. Each ss motif profile is represented by a 4 × 9 or a 4 × 14 PWM.

### Calculation of information score of a feature

From information theory, the amount of information held by a given motif is related to the relative entropy of the motif. Meanwhile, the likelihood of a given sequence belonging to the motif is estimated from the log-odds scores. Most importantly, log-odds can measure the amount of "information for discrimination" between the motif and the background [45]. In general, the higher the log-odds of a motif are, the rarer the occurrence of similar sequences in the background is. We evaluated the information score of each of the five features (5'ss, 3'ss, BP, intron length, and nucleotide composition within an intron) of an intron by this standard method.

### 5'ss, 3'ss, and BP

For 5'ss, 3'ss, and BP, the log-odds are tabulated in the form of PWM:

(2) pwm(k,j)=log2((fk,j+ε)(Fk+ε))

where *f*_*k,j *_is the relative frequency of observing nucleotide *k *at the motif position *j*, *F*_*k *_is the background frequency of *k*, and *ε *is a small constant representing a pseudo count (10^-4 ^by default). Note that we numerically encoded nucleotides A, C, G, and T into 1, 2, 3, and 4, respectively, and 1 ≤ *j *≤ *w*, where *w *denotes the number of columns of PWM. As mentioned above, *w *= 9 for 5'ss, *w *= 14 for 3'ss, and *w *= 7 for BP in this study.

Given a segment of genomic nucleotide sequence, **s**(*i*:*i *+ *w*-1) = *s*_*i*__,_*s*_*i*__+ 1_,...,*s*_*i*__+ __*w*__-1_, the information (log-odds) score *IS*_feature_(**s**(*i*:*i *+ *w*-1)) of the segment having a feature (5'ss, 3'ss, or BP) is defined as:

(3) $$IS_{\text{feature}}\left(s\left(i:i+w-1\right)\right)=\sum_{j=1,w}pwm\left(s_{i+j-1},j\right).$$

### Intron length

The distribution of intron lengths, *f*(*l*), is characteristic to each species (see Figure 6 for the results of six species). We modeled the distribution of intron lengths in each species by the superposition of two Frechet distributions [27], and measured the information content assigned to an intron of length *l *(*L*_min _≤ *l *<*L*_max_) by a simple log-odd:

(4) ISintorn_length(l)=log2(f(l)c).

As the background, we used a uniform density of *c *= 1/(*L*_max _- *L*_min_). *L*_min _was fixed to 30, whereas *L*_max _was determined by the 99% quantile of the longer components of the Frechet distributions.

### Oligomer composition within intron

Although the coding potential is widely used in gene recognition programs, the usual coding potential is beyond the scope of this study as our present interest is in intron recognition. Although less remarkable than coding sequences, intronic sequences still have some compositional bias relative to the whole genomic sequences. We modeled both intronic and whole genomic sequences by the homogeneous fourth-order Markov models, which are nearly equivalent to accounting 5-mer frequencies. Given a nucleotide sequence **s **of a potential intron, we applied the Markov models to **s **to calculate the probability of **s **having the intronic composition, *P*_I_(**s**), and the probability of **s **having the background composition, *P*_G_(**s**). Then, we defined the information score on the oligomer composition of an intron of length *l *as

(5) IScomposition(S)=log2(PI(S)PG(S))/l

### Calculation of information content of each motif

The information content (*IC*) of a motif with a distribution *f *is defined as usual by the relative entropy against the background distribution *g*:

(6) $$IC_{\text{motif}}=\sum_{m}f_{m}\log_{2}\left(f_{m}/g_{m}\right),$$

where *f*_*m *_is the probability of observing sequence *m *under the motif distribution, *g*_*m *_is the probability of observing sequence *m *under the background sequence distribution, and the sum is taken over all possible nucleotide sequences of the motif length.

### Calculation of distance and construction of feature trees

We calculated the distance between a pair of species, *A *and *B*, concerning each of the five features, *i.e.*, the three kinds of motif profiles, the intron length, and the oligomer composition within an intron. Let **f **and **g **be matrices or vectors that characterize a feature of species *A *and *B*, respectively. By **F **and **G**, we denoted the corresponding background probabilities obtained from the whole genomic sequences of *A *and *B*, respectively.

For a motif profile of 5'ss, 3'ss or BP, **f **or **g **is represented by a 4 × *w *matrix, in which the columns corresponding to the terminal dinucleotides of introns were omitted from calculation of the distance between the two motif profiles of 3'ss or 5'ss. We slightly modified the symmetric Kullback-Leibler divergence [46] to measure the distance *D*_feature_(*A*, *B*) between *A *and *B *concerning a certain feature as follows:

(7) Dfeature(A,B)=∑i=1,w ∑k=1,4{fk,ilog2(fk,iGkFkgk,i)+gk,ilog2(Fkgk,ifk,iGk)}.

For simplicity, we omitted the terms corresponding to pseudo counts here and below. The first term in the braces of equation (7) can be rewritten as:

(8) fk,j{log2(fk,jFk)−log2(gk,jGk)},

which indicates that the value is the difference in the expected motif score for species *A *calculated with the "cognate" log-odds PWM and that calculated with the heterogeneous log-odds PWM. The second term has the same meaning for species *B*. Note that *D*_feature_(*A*, *B*) defined above and *D*_composition_(*A*, *B*) defined below are not guaranteed to be non-negative and so do not satisfy the axiom of distance. However, they have a straightforward meaning as a measure of divergence, and work fine in practice.

For oligomer compositions, **f **or **g **is the probability of occurrence of a 5-mer within introns, and **F **or **G **is the corresponding probability observed in the whole genomic sequence. Similar to equation (7), we defined the distance as:

(9) Dcomposition(A,B)=∑m{fmlog2(fmGmFmgm)+gmlog2(FmgmfmGm)},

where the summation was taken for all 5-mers.

To calculate the distance between the distributions of intron lengths of two species, we defined *f*(*l*) or *g*(*l*) as the frequency fitted to two Frechet distributions of length *l*. We evaluated the distance by the symmetric Kullback-Leibler divergence:

(10) Dintron_length(A,B)=∑l{f(l)log2(f(l)g(l))+g(l)log2(g(l)f(l))},

where the summation was taken over the range: *L*_min _≤ *l *< Min(*L*_max_(*A*), *L*_max_(*B*)).

Using the distance matrices separately derived for the five features, we generated UPGMA (Unweighted Pair Group Method with Arithmetic mean) trees [47] by the "neighbor" program in the *Phylip *package [48].

As controls, we also constructed phylogenetic trees from 18S rRNA and U2 snRNA sequences. We collected a set of 18S rRNA sequences of the 61 species from Silva [49] and NCBI. U2 snRNA sequences were obtained from several sources: Rfam [36], [23], [50], and NCBI. When multiple copies of 18S rRNA or U2 snRNA genes were present in a species, only the one closest to the consensus was selected. We aligned the RNA sequences by Mafft [51,52] with the Q-INS-I option, and then generated phylogenetic trees by using four methods (UPGMA, neighbor-joining, maximum parsimony, and maximum likelihood) implemented in the *Phylip *package [48]. In this study, however, we report only the results with the maximum likelihood method.

### Evaluation of degree of discordance between a feature tree and reference tree

To evaluate how much a feature tree deviates from a reference tree, we used the nodal distance method implemented in TOPD/FMTS v3.3 [53]. In this method, the number of internal nodes that intervene between every pair of taxa in a given tree is counted to generate the nodal distance matrix. Then, the distance between each feature tree and each reference tree is evaluated by the root mean squared deviation (RMSD) between the corresponding elements of the two nodal distance matrices. An RMSD value of zero indicates that the two trees being compared are identical, while a larger RMSD value indicates greater discordance between the trees under comparison. This software also provides a tool for statistical test, which generates a number of (100 by default) trees with the same taxa and the same topology as those of the original tree but the labels of the taxa are randomly permutated. By applying this method to each of the five feature trees, we obtained the mean and standard deviation of RMSD values for the randomized trees. Assuming a normal distribution with these statistics, we estimated the significance of relatedness between the observed feature tree and the reference tree, and present the results in the caption of Figure 4. For each feature tree, we evaluated the discordance score with the mean of RMSD values obtained with the 18S rRNA tree and the U2 snRNA tree as controls.

### Evaluation of relative contribution of each feature

We employed a method similar to that proposed by Lim and Burge [5] to evaluate the relative contributions of the five features discussed in the subsection "Calculation of Information Score of a Feature."

For a given intron, we enumerated all possible 5' and 3' splicing signal pairs within the region from 100 bp upstream of the 5'ss to 100 bp downstream of the 3'ss, besides the true one, which follow the canonical GY-AG or AT-AC rule and are separated by typical lengths of short introns, and assigned them the "splicing score," which is defined as the sum of information scores of the five features. On average, 393 pairs were examined for each intron. We chose the pair with the highest splicing score as the predicted intron boundaries. The intron detection accuracy is defined as *Ac *= (the number of correctly predicted introns)/(the total number of real introns examined). We regarded a prediction to be successful if *Ac *exceeded 0.98 [5].

We measured the contribution of each feature to intron recognition as follows. First, we examined the accuracies of intron recognition for all combinations of features involving both splicing signals, 5'ss and 3'ss. For example, 5'ss + 3'ss, 5'ss + 3'ss + BP, 5'ss + 3'ss + length + composition, etc. Next, we transformed the accuracy to *TAc *= -log(1.0-*Ac*). We defined the "necessary amount of contribution" as *TAc *of accuracy 98% (-log(1.0-0.98=0.02)). The difference between the necessary amount of contribution and the maximal *TAc *is defined as the information deficit for intron recognition. Each contribution of the five features is defined as the difference in the amount of contribution corresponding to the gain in accuracy obtained with 5'ss + 3'ss + the feature from that with the minimal *TAc*. For example, the information value of BP is obtained by subtracting *TAc *of 5'ss + 3'ss from *TAc *of 5'ss + 3'ss + BP. When the gain is negative, the corresponding amount of contribution is zero. Finally, we calculated the ratio of each feature's *TAc *to the necessary amount of contribution and defined the ratio as the "information share" of the feature.

### Clustering method

We tried to classify all the 61 species according to the contributions of the five features plus the fraction of "information deficit." In the method, we first applied the PCA to the contribution data to obtain the first and second components. We used the raw contribution values of the six features as the input to PCA. Using these PCA components, we classified the 61 species by the *k*-means clustering method [54,55]. As a preliminary result of the SOM classification of the same data indicated that the 61 species were divided into six clusters (data not shown), we used six as the number of clusters with the *k*-means clustering method.

## List of abbreviations

BP: branch point; PCA: principal component analysis; PPT: polypyrimidine tract; PWM: position-specific weight matrix; SOM: self-organization map; ss: splice site.

## Authors' contributions

HI carried out the majority of the analyses and drafted the manuscript. OG conceived of the study, carried out a part of the analyses, and helped to draft the manuscript. Both authors read and approved the final manuscript.

## Supplementary Material

Additional file 1 **Synopsis of splice sites identified**. This table shows the numbers and percentages of individual subtypes as well as the total numbers and the average lengths of introns identified by our procedure for each species. The numbers of short introns and their fractional percentage are also presented. Click here for file

Additional file 2 **Three PPT signals of the 61 species presented in the descending order of strength**. (a) Percentages of introns in which PPTs are detected. (b) Average lengths of identified PPTs. (c) Percentages of C + T content within PPT. Color codes are identical to those in Figures 4 and 7. Click here for file

Additional file 3 **Characteristics of PPTs**. a) Percentages of introns in which PPTs are detected. b) Average lengths of identified PPTs. c) Average percentages of pyrimidine nucleotides in PPTs. Click here for file

Additional file 4 **Information contents in bits for the three splicing signal motifs**. Click here for file

Additional file 5 **Relative contributions of the five features and the information deficit to short introns recognition**. The difference between the necessary amount of contribution and the maximal *TAc *is defined as the information deficit for intron recognition (see the subsection of Evaluation of Relative Contribution of Each Feature in Methods). The color codes are identical to those in Figures 4 and 7. Click here for file

Additional file 6 **Contribution of the five features to short introns recognition and information deficit**. Click here for file

Additional file 7 **PCA plots of four main components**. The plots show correlations between (a) the first and the second principal components, (b) the first and the third principal components, and (c) the first and the fourth principal components. The proportion of variance of the first, second, third and fourth principal components are 47.2%, 25.4%, 16.0% and 6.7%, respectively. Click here for file

Additional file 8 **Statistics of AT-AC introns**. The total number of AT-AC introns, the number of AT-AC introns with 5'ss motif score greater than 9 bits, that with BP motif score greater than 6 bits, that with intron length < 20 kb, and that of U12-type introns (that satisfy these three criteria) in five representative species. Click here for file
