# Supplementary figures and images for: Comparative analysis of information contents relevant to recognition of introns in many species
Source: BMC Genomics. 2011 Jan 19;12:45. doi: 10.1186/1471-2164-12-45 (PMC3033335; doi:10.1186/1471-2164-12-45)

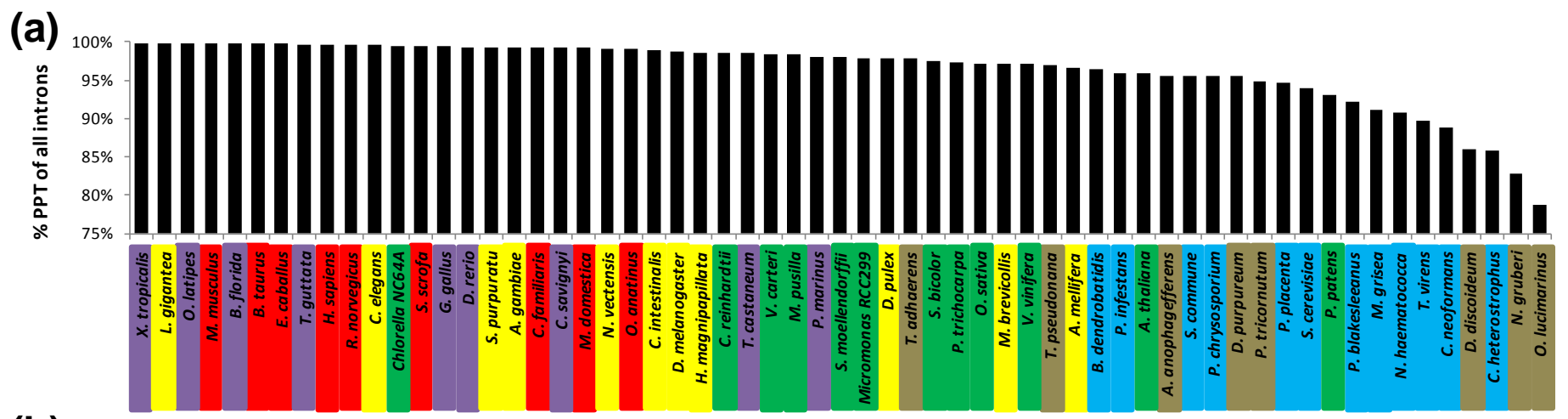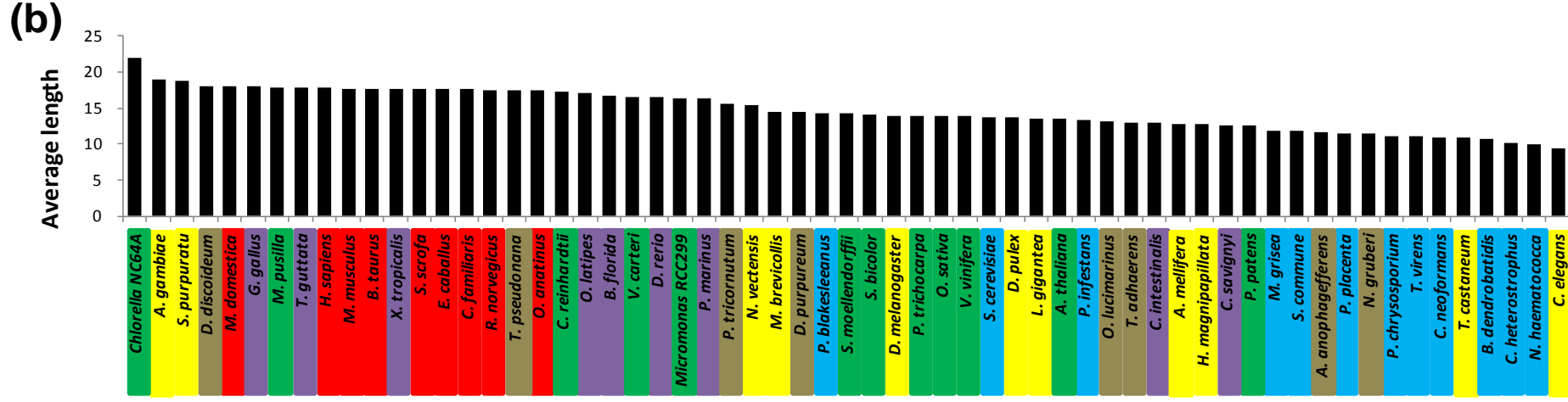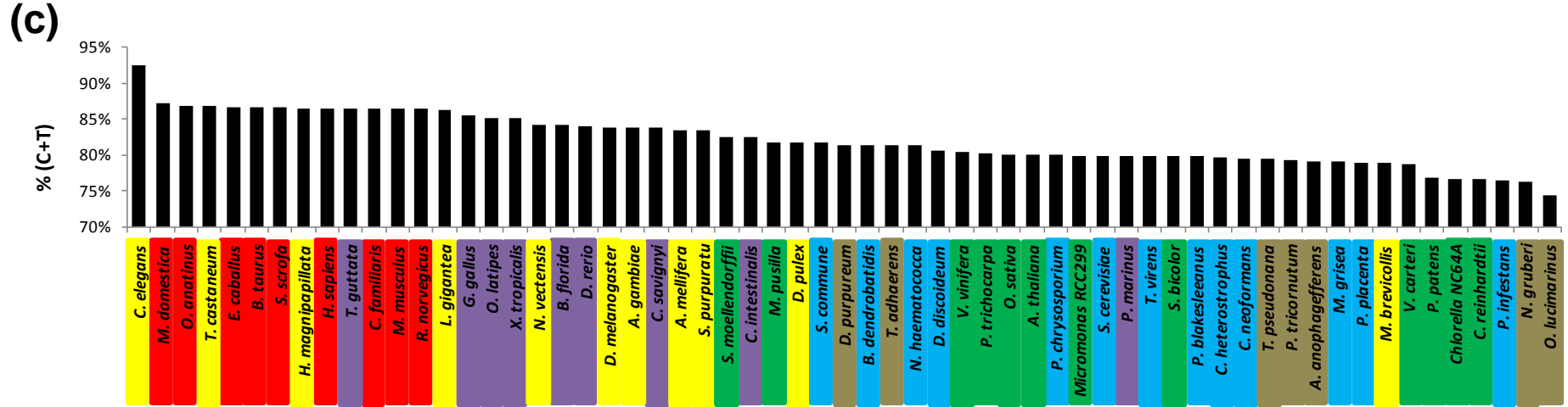

Supplement: Additional file 2 — Three PPT signals of the 61 species presented in the descending order of strength. (a) Percentages of introns in which PPTs are detected. (b) Average lengths of identified PPTs. (c) Percentages of C + T content within PPT. Color codes are identical to those in Figures 4 and 7. [file 1471-2164-12-45-S2.PDF]

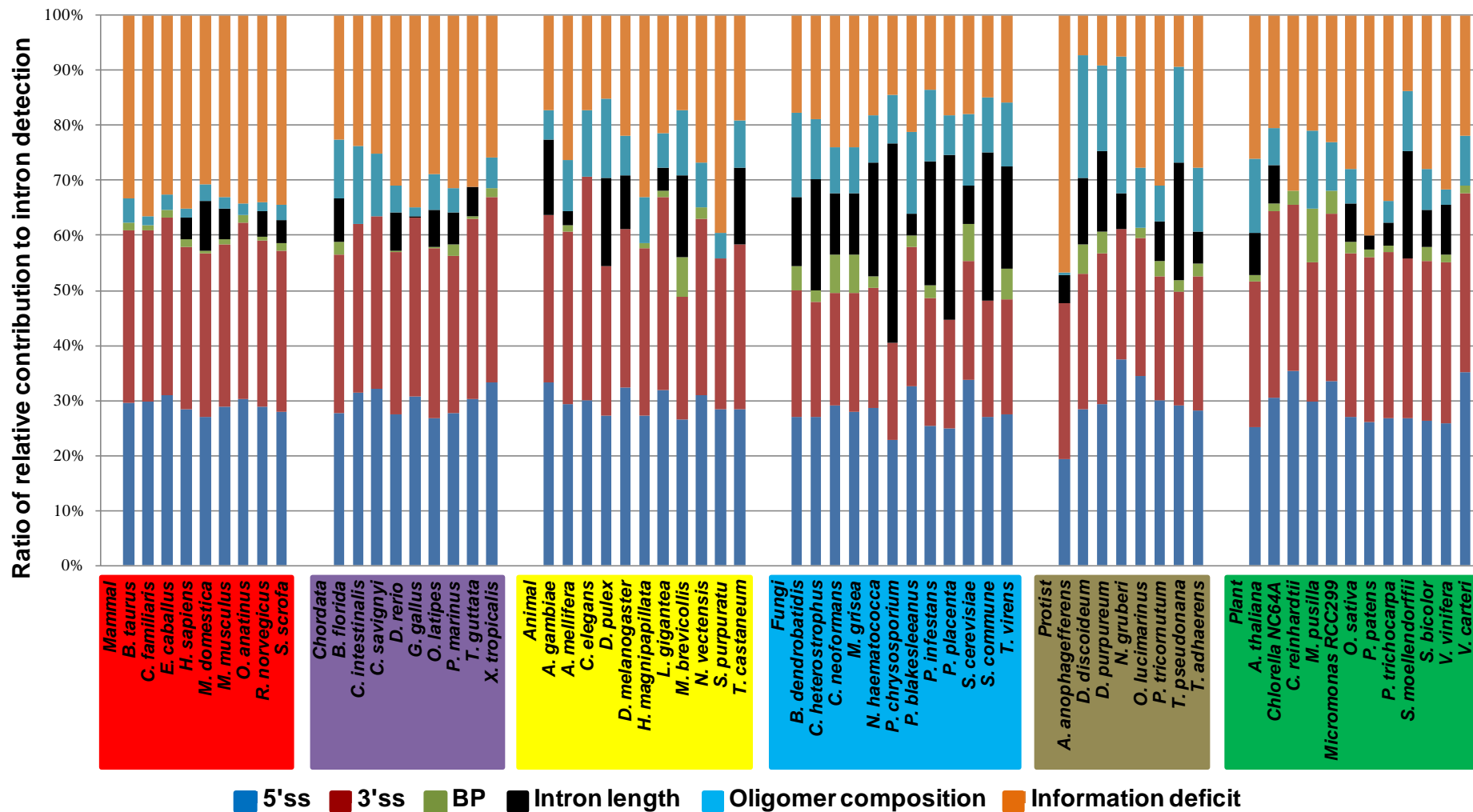

Supplement: Additional file 5 — Relative contributions of the five features and the information deficit to short introns recognition. The difference between the necessary amount of contribution and the maximal TAc is defined as the information deficit for intron recognition (see the subsection of Evaluation of Relative Contribution of Each Feature in Methods). The color codes are identical to those in Figures 4 and 7. [file 1471-2164-12-45-S5.PDF]

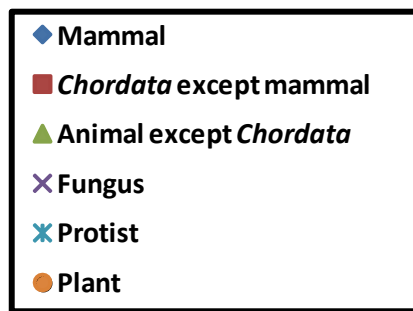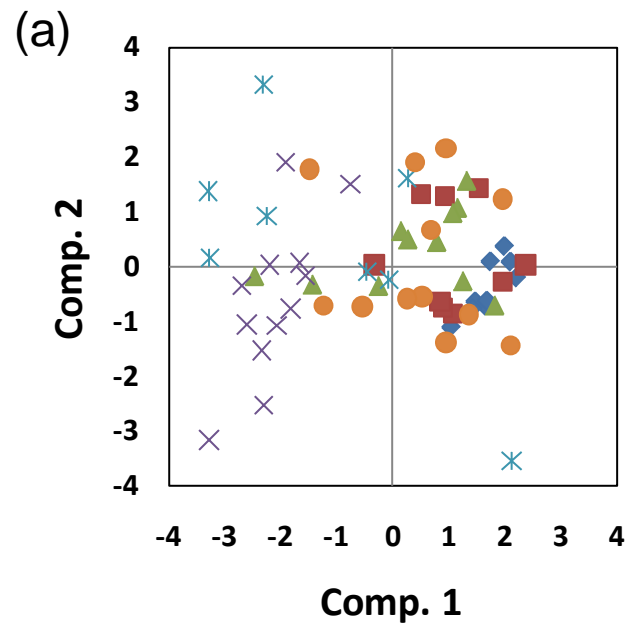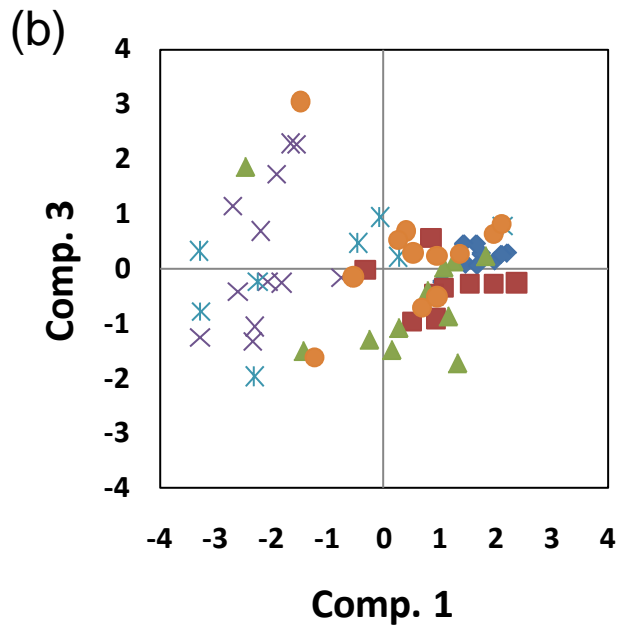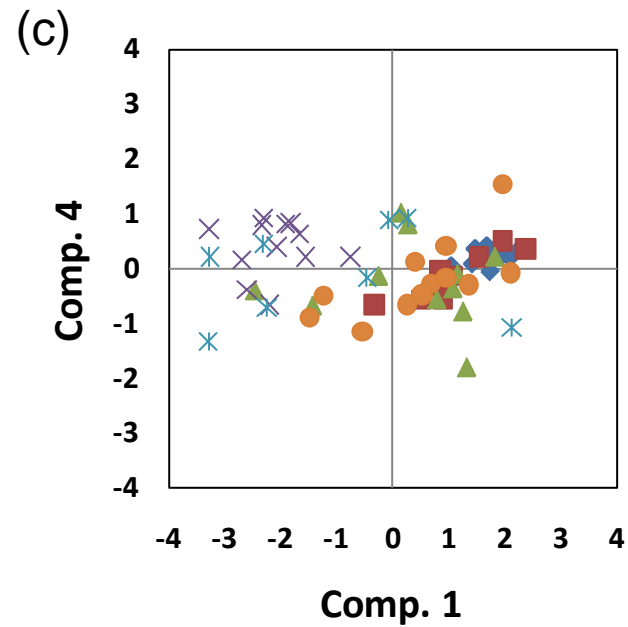

Supplement: Additional file 7 — PCA plots of four main components. The plots show correlations between (a) the first and the second principal components, (b) the first and the third principal components, and (c) the first and the fourth principal components. The proportion of variance of the first, second, third and fourth principal components are 47.2%, 25.4%, 16.0% and 6.7%, respectively. [file 1471-2164-12-45-S7.PDF]
